# Macrophage Functions and Regulation: Roles in Diseases and Implications in Therapeutics

**DOI:** 10.1155/2018/7590350

**Published:** 2018-06-05

**Authors:** Kebin Hu, Yang Jin, Zissis Chroneos, Xiaodong Han, Hao Liu, Ling Lin

**Affiliations:** ^1^Department of Cellular and Molecular Physiology, The Pennsylvania State University College of Medicine, Hershey, PA, USA; ^2^Division of Nephrology, Department of Medicine, The Pennsylvania State University College of Medicine, Hershey, PA, USA; ^3^Department of Medicine, Boston University School of Medicine, Boston, MA, USA; ^4^Department of Pediatrics, Microbiology and Immunology, The Pennsylvania State University College of Medicine, Hershey, PA, USA; ^5^Immunology and Reproduction Biology Laboratory & State Key Laboratory of Analytical Chemistry for Life Science, Nanjing University Medical School, Nanjing, Jiangsu, China; ^6^Department of Neurology, University of Pittsburgh School of Medicine, Pittsburgh, PA, USA

Macrophages, as a key element in innate immunity, play an important role in the first-line defense against pathogens and modulating inflammatory responses. From the traditional point of view, tissue macrophages are differentiated from bone marrow myeloid progenitor-derived monocytes in the circulation and undergo a fine-regulated process of adaption to the local tissue microenvironment [1]. However, over the past decade, mounting evidence has demonstrated that macrophages are also derived from embryonic York sac and fetal liver and become a self-maintaining population residing locally and performing organ-specific functions [2].

Macrophages are not homogenous and consist of variably mixed populations, such as liver Kupffer cells and brain microglial cells that carry out specific functions in the local microenvironment [3]. In response to various physiological or pathological cues, macrophages display an extended life span and acquire different functional phenotypes through polarization that are generally categorized into two broad but distinct subsets as either classically activated (M1) or alternatively activated (M2). In general, M1 macrophages have high motility and promote inflammation and damage through a combination of transcription factors such as NF-*κ*B, whereas M2 macrophages help to resolve inflammation and promote tissue remodeling [4]. Notably, M1 and M2 only represent two extremes of macrophage polarization, and most differentiated macrophages fall into a full spectrum of various polarization states between M1 and M2. In addition, macrophage polarization is a dynamic process and macrophages can switch their phenotypes between M1 and M2 in different pathological conditions [5]. Nevertheless, sustained macrophage infiltration in face of injury eventually becomes pathological and causes distorted repair and remodeling, leading to irreversible tissue destruction and disease progression and deterioration. Thus, better understanding of the regulation of macrophage differentiation and polarization, as well as their roles in disease pathogenesis, will contribute to the development of selective and effective therapies.

In this specific issue, fourteen quality manuscripts were selected for publication from a large number of submissions covering various topics of macrophage functions and regulation, as well as their roles in diseases and therapeutics. Ten of these publications are review articles reflecting the current status of knowledge and advances in understanding macrophage functions and regulation. L. Parisi et al. provided a comprehensive review regarding the role and regulation of M1-like (killers) and M2-like (builders) macrophages in various chronic diseases including cancers, type 2 diabetes, atherosclerosis, and periodontitis. They also discussed therapeutic approaches using cytokine antagonists and miRNAs. J. Yin et al. highlighted the current understanding of microglia and macrophage functions and differentiation in CNS homeostasis, autoimmunity, and cancer. Other review articles are more focused with emphasis on an individual disease, molecule, or pathway. J. Shi et al. discussed the roles of macrophage subsets in bowel anastomotic leakage and healing. L. Shao et al. summarized the perspectives and potential targets of macrophage polarization in cerebral aneurysm, and L. Zhu et al. reviewed the roles of members of the phospholipase C family in macrophage-mediated inflammation. T.S. Kapellos et al. updated the current knowledge regarding macrophage dysfunction in chronic obstructive pulmonary disease with a focus on the well-known resident alveolar macrophages, whereas, J. Schyns et al. illuminated the important role of the less-studied lung interstitial macrophages. Of note, there are two review articles about macrophage tolerance and regulation from R. Huber et al. and R. Ocaña-Guzman et al. with focus on TNF and inhibitory receptors, respectively. H. Liao et al. provided an interesting retrospective literature analysis regarding the role of macrophage iNOS activity in the therapeutic effect of Huangqi, a traditional Chinese medicine, on diabetic nephropathy. The remaining four accepted manuscripts are research articles of translational significance using various patient samples or animal models to study macrophage functions and regulation. M. Yamashita et al. examined the expression pattern of CD163-positive macrophages in lung biopsy samples from patients with idiopathic interstitial pneumonias. I.A. da Silva et al. investigated the role of the platelet-activating factor in modulating the tumor-associated macrophage phenotype, and W.R. Shen et al. demonstrated the potential therapeutic role of targeting osteoclast formation in LPS-induced bone loss. Y.M. Flores-Martinez et al. established a rat model of Parkinson's disease with classical microglia activation, neuroinflammation, and degeneration. These research articles all highlighted the important role of macrophage functions and regulation in disease pathogenesis and therapeutics.

In summary, these articles illuminate the role and regulation of macrophage function and differentiation in the pathogenesis and therapeutics of various diseases, and provide guidance for future research on macrophage functions and development of selective and efficient therapeutics.

*Kebin Hu* *Yang Jin* *Zissis Chroneos* *Xiaodong Han* *Hao Liu* *Ling Lin*
